# Impact of Shiga-toxin encoding gene transduction from O80:H2 Shiga toxigenic *Escherichia coli* (STEC) on non-STEC strains

**DOI:** 10.1038/s41598-022-26198-8

**Published:** 2022-12-14

**Authors:** Audrey Habets, Céline Antoine, Jeroen Wagemans, Marjorie Vermeersch, Fanny Laforêt, Jacob Diderich, Rob Lavigne, Jacques Mainil, Damien Thiry

**Affiliations:** 1 Bacteriology, Department of Parasitic and Infectious Diseases, FARAH, ULiège, 4000 Liège, Belgium; 2 Laboratory of Gene Technology, Department of Biosystems, KU Leuven, 3000 Leuven, Belgium; 3 Electron Microscopy Laboratory, Center for Microscopy and Molecular Imaging, ULB, 6041 Gosselies, Belgium

**Keywords:** Bacteriophages, Bacterial pathogenesis

## Abstract

Shiga toxin-producing *Escherichia coli* (STEC) are major foodborne pathogens that cause human diseases ranging from diarrhea to life-threatening complications including hemolytic–uremic syndrome. Virulence of STEC strains and their ability to cause severe diseases are associated with the activity of prophage-encoded Shiga toxins (Stxs). The first objective of this work was to isolate and characterize the Stx2d phage from STEC O80:H2 and to study the transfer of this phage in non-STEC strains. The second objective was to assess the survival of *Galleria mellonella* larvae inoculated with these transduced strains. Firstly, one bacteriophage isolated from a STEC O80:H2 strain was used to infect six non-STEC strains, resulting in the conversion of three strains. Then, stability assays were performed, showing that this phage was stable in the new STEC strains after three successive subculturing steps, as confirmed by a combination of short and long read genome sequencing approaches. This phage, vB_EcoS_ULI-O80_Stx2d, is resistant to moderate temperature and pH. It belongs to a currently unclassified genus and family within the *Caudoviricetes* class, shares 98% identity with Stx2_112808 phage and encodes several proteins involved in the lysogenic cycle. The *yecE* gene was identified at the insertion site. Finally, *G. mellonella* experiments showed that the transduced strains caused significantly higher mortality rates than the corresponding non-STEC strains. In conclusion, this study showed that *stx2d* gene from O80:H2 *E. coli* can be transferred to non-STEC strains and contributes to their virulence.

## Introduction

Shiga toxin (Stx)-producing *Escherichia coli* (STEC) are notorious foodborne pathogens that cause several human diseases, such as diarrhea, hemorrhagic colitis and hemolytic uremic syndrome (HUS)^1^. Shiga toxin (Stx), encoded by Stx-converting bacteriophages (Stx phages), is one of the primary virulence factors of STEC strains and can exhibit cytotoxicity to various target cells^2,3^. Stx toxins belong to two families (Stx1 and Stx2). Each of these types are further divided into subtypes, three for Stx1 (Stx1a, Stx1c and Stx1d) and at least seven for Stx2 (Stx2a, Stx2b, Stx2c, Stx2d, Stx2e, Stx2f and Stx2g)^4^, although new Stx2 subtypes are regularly described, including Stx2h to m^5^. The *stx1* and *stx2* genes which encode these toxins are contained within temperate bacteriophage genomes of the former *Podoviridae, Myoviridae* or *Siphoviridae* families^3^, integrated as prophages within the bacterial chromosome*.* The Stx phages ability to excise themselves and infect other hosts, e.g. in the gastrointestinal tract, makes them important drivers of horizontal gene transfer (HGT) of *stx* genes among *E. coli* serotypes and other *Enterobacteriaceae*. This ability to quickly gain, lose or exchange genes through Stx phages impacts the pathogenicity profile and evolution of STEC strains. Integrated *stx* genes either remain silent within the lysogens^6^ or are expressed at low levels following infection with a Stx-converting phage.

STEC can be exposed to various stresses, including heat, mitomycin C, and UV, leading to a bacterial S.O.S response. This response is characterized by the excision of the prophage DNA from the host chromosome and the initiation of the lytic cycle^7^. This results in host cell lysis and the release of phage particles that can transduce the *stx* genes to other bacteria^8–10^.

Nine Stx phage insertion sites have been described to date, including *wrbA*, which encodes for a tryptophan repressor-binding protein^11^, *yehV* encoding a transcriptional regulator^12^ and *yecE*, whose function is unknown^13^.

Evidence suggests that STEC O157:H7 is responsible for numerous outbreaks and has evolved from an EPEC O55:H7 strain by acquisition of Stx2c phage followed by multiple introductions of Stx2a and Stx1a phages^14^. Other studies have shown that the enteroaggregative STEC strain (EAEC) O104:H4, responsible for the German epidemic in 2011, was driven by the Stx phage transfer from a STEC strain^15^. Stx phage transfers have also been demonstrated in vitro and in vivo for specific classic serotypes, such as O26:H11, after extraction of the phage from the donor bacterium^9,16^. Over the last 10 years, a novel serotype of STEC, O80:H2, has emerged as a cause of STEC-HUS^17^. This serotype has been isolated from patients with STEC-HUS presenting multi-organ failure, and extensive thrombotic microangiopathy^18^. STEC O80:H2 has been described as a hybrid pathotype that combines the diarrhoeagenic *E. coli* virulence factors (VFs) Stx, Eae (intimin) and EhxA (enterohaemolysin), with extra-intestinal VFs^19^.

Initially described in France^20^, serotype O80:H2 emerged in the 2010s to become one of the three leading serotypes involved in HUS cases^21^. Moreover, this serogroup has also been isolated from cattle in Spain^22^, humans and cattle in Belgium^23–25^, human in Switzerland^26^ and the Netherlands^18^. It now represents the third most frequent serotype isolated from HUS cases in Europe, responsible for 9% of European HUS in 2019^27^.

It has been hypothesized that EPEC O80 is the ancestor of STEC O80^28^. According to this hypothesis, the acquisition of *stx2* by EPEC O80 gave rise to globally distributed toxigenic STEC O80. Historically, epidemiological studies have focused on *stx2*-harboring strains because they are typically associated with more severe clinical outcomes^29,30^.

A recent study highlighted the circulation of EPEC strains of the same serotype in diarrheic calves^25^. Genomic analysis showed that the bovine O80:H2 EPEC are closely associated to human O80:H2 STEC but contain neither the *stx* genes nor the Stx phages. It is therefore possible that the bovine EPEC strains were AE-STEC that lost the Stx phage or represent precursors of AE-STEC which have acquired the Stx phage.

The objective of this work was to isolate and characterize the Stx2d phage from STEC O80:H2, to study the STX phage transfer in non-STEC strains and to assess the virulence of the newly converted strains in vivo in a *Galleria mellonella* larvae model.

## Results

### Isolation, host range analysis of Stx2d phages and horizontal transfer of *stx2d* gene from O80:H2 *E. coli* to non-pathogenic *E. coli*

Three temperate phages were induced and isolated from three bovine O80:H2 STEC strains by UV radiation. Lytic plaque formation was analyzed to assess the ability of Stx2d phages to infect a range of host strains (Table 1). The host range analysis showed that the three Stx2d phages isolated from O80:H2 donor strains EH3155, EH3160 and EH3320 produced plaque lysis on strains K12-MG1655, K12-DH5α, O80:H26 and O80:H2.

**Table 1:** *Escherichia coli* strains used in this study.

Bacterial strains	*stx* gene	Serotype	References
**Donor strains**			
EH3155	*stx2d*	O80:H2	^23^
EH3160	*stx2d*	O80:H2	
EH3320	*stx2d*	O80:H2	
**Host (recipient) strains**			
K12-MG1655	–	–	Laboratory strain
K12-DH5α	–	–	Laboratory strain
ATCC700973	–	O18:K1	ATCC
ATCC25922	–	–	ATCC
O80:H2	–	O80:H2	Laboratory strain
O80:H26	–	O80:H26	^25^

The three phages were used to transduce these four positively lysed strains. Three transduced strains (K12-MG1655, K12-DH5a, O80:H26) were confirmed by PCR targeting the *stx2d* gene*.* A stability study indicated that the phage isolated from the strain EH3320 was stable in the new STEC strains after three successive subculturing steps unlike the two others (EH3155 and EH3160). This phage was therefore selected for further analysis.

### Genome-based characterization of vB_EcoS_ULI-O80_Stx2d confirms this phage as a temperate lambda-like virus

**Figure 1:**
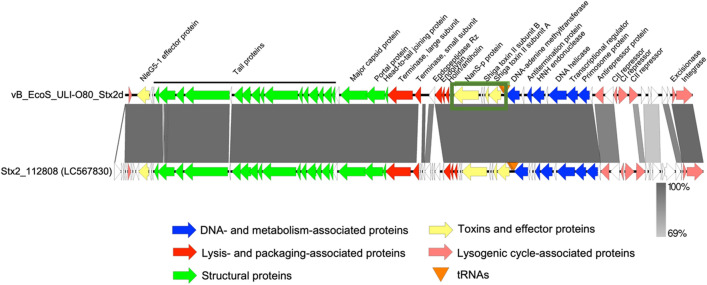
Comparative genomics of phage vB_EcoS_ULI-O80_Stx2d and Stx2_112808. Each arrow represents a coding sequence. In red, genes coding packaging and lysis-associated proteins are displayed; in green, structural proteins; in blue, DNA and metabolism-associated proteins; in pink, lysogenic-associated proteins. The *stx* region is highlighted in a box with toxins and effectors proteins like the two Shiga toxin II subunit A and B and the the *nanS-p* gene represented in yellow.

This Stx2d phage was analysed by whole genome sequencing, revealing a dsDNA genome of 40,147 bp (Fig. 1). The phage was named according to the standardized phage nomenclature vB_EcoS_ULI-O80_Stx2d, belongs to the *Caudoviricetes* class and a yet unclassified family and genus (Supplementary Fig. 1). The closest similar virus is phage Stx2_112808 (LC567830.1, 88% query coverage, 98.03% sequence identity) and vB_EcoS_ULI-O80_Stx2d shares > 95% identity with seven other phages according to BLASTn analysis. Homology detection by BLASTp and structure prediction by HHpred analysis^31^ revealed that the two phages encode several lysogeny associated proteins including an integrase (Int), excisionase (Xis) and different repressors like CI, CII and the antirepressor Cro involved in maintaining the lysogenic cycle. Comparison of phage genomes are shown in Fig. 1. The “stx region”, defined as the phage’s genome region encompassed by the genes Q and S and containing the Stx-coding genes was localized. Noteworthy, the presence of nanS-p gene exclusively in the “stx region” of the phage identified (Fig. 1). Sequencing was submitted to NCBI GenBank under accession number ON416862.

### Bacteriophage characterization

**Figure 2:**
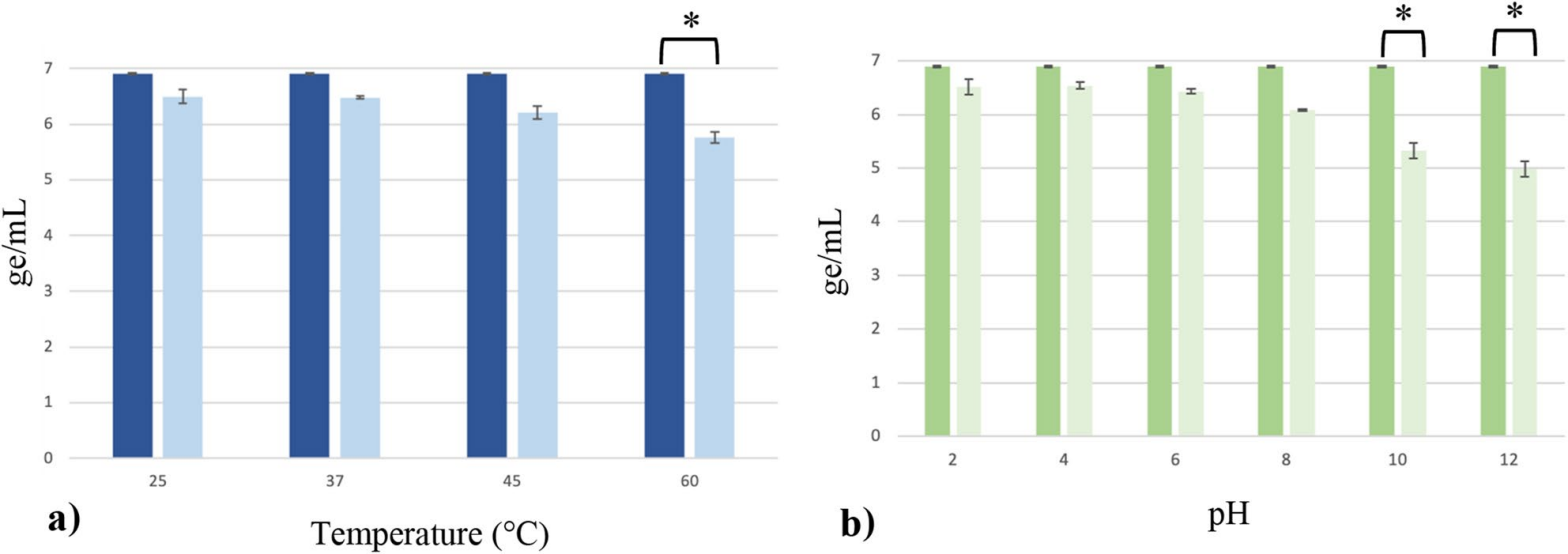
(**a**) Temperature (**b**) pH stability results of phage vB_EcoS_ULI-O80_Stx2d after 1 h. The means of three experiments (± σ) are represented and the concentrations measured before testing are represented by the dark bars. *p* value (*) ≤ 0.05. *ge* genomic copies.

The phage vB_EcoS_ULI-O80_Stx2d isolated from the EH3320 strain was first characterized for its pH and temperature stability. This one remained stable (< 1 ge/mL) at temperatures of 25 °C, 37 °C and 45 °C. At 60 °C, the phage showed a 1 ge/mL reduction (Fig. 2a). Concerning the pH stability, the phage remained stable between pH 2 and pH 8 and began to decrease with 1 ge/mL at pH 10 and 2 ge/mL at pH 12 (Fig. 2b). This phage thus presented resistance to moderate temperature (45 °C) and a broad pH spectrum (2–8).

**Figure 3:**
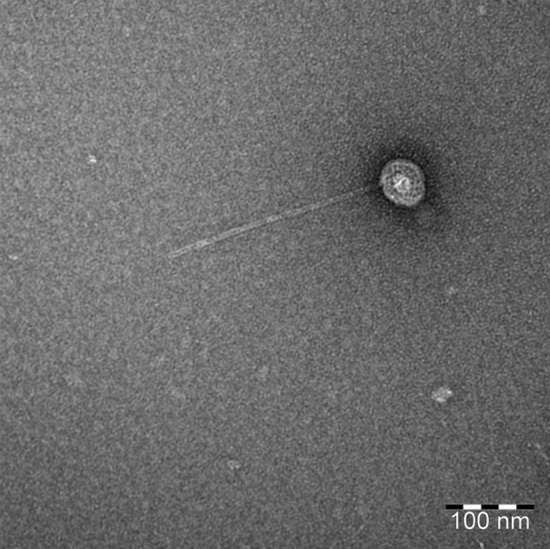
Negative staining transmission electron microscopic image of the phage vB_EcoS_ULI-O80_Stx2d.

The vB_EcoS_ULI-O80_Stx2d morphology was also characterized by Transmission Electron Microscopy (TEM). This phage presented an icosahedral symmetric non-enveloped head, with a diameter of approximately 60 nm, and a non-contractile and flexible tail of approximately 245 nm in length corresponding to a siphovirus morphology (Fig. 3).

### Insertion sites of the phage vB_EcoS_ULI-O80_Stx2d

**Figure 4:**
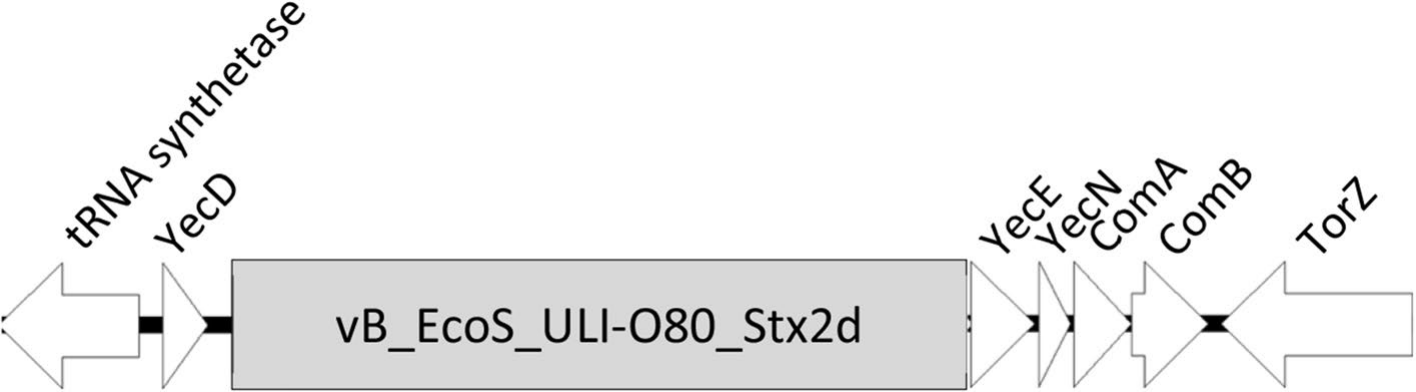
Insertion site *yecE* of the three transduced strains. tRNA synthetase: Aspartyl-tRNA synthetase, YecD: isochorismatase-family protein, YecN: Inner membrane protein YecN, ComA: Carboxy-S-adenosyl-l-methionine synthase, ComB: tRNA ho5U(34) carboxymethyltransferase, Tor-Z: Trimethylamine-*N*-oxide reductase.

Bacterial genomic analysis were performed in order to highlight the presence and the type of insertion sites. The identified insertion site of vB_EcoS_ULI-O80_Stx2d into the chromosome of the three transduced strains was the same as within the donor strains: the *yecE* gene (Fig. 4).

### Assessing the in vivo virulence properties of the prophage in the *Galleria mellonella* larvae model

**Figure 5:**
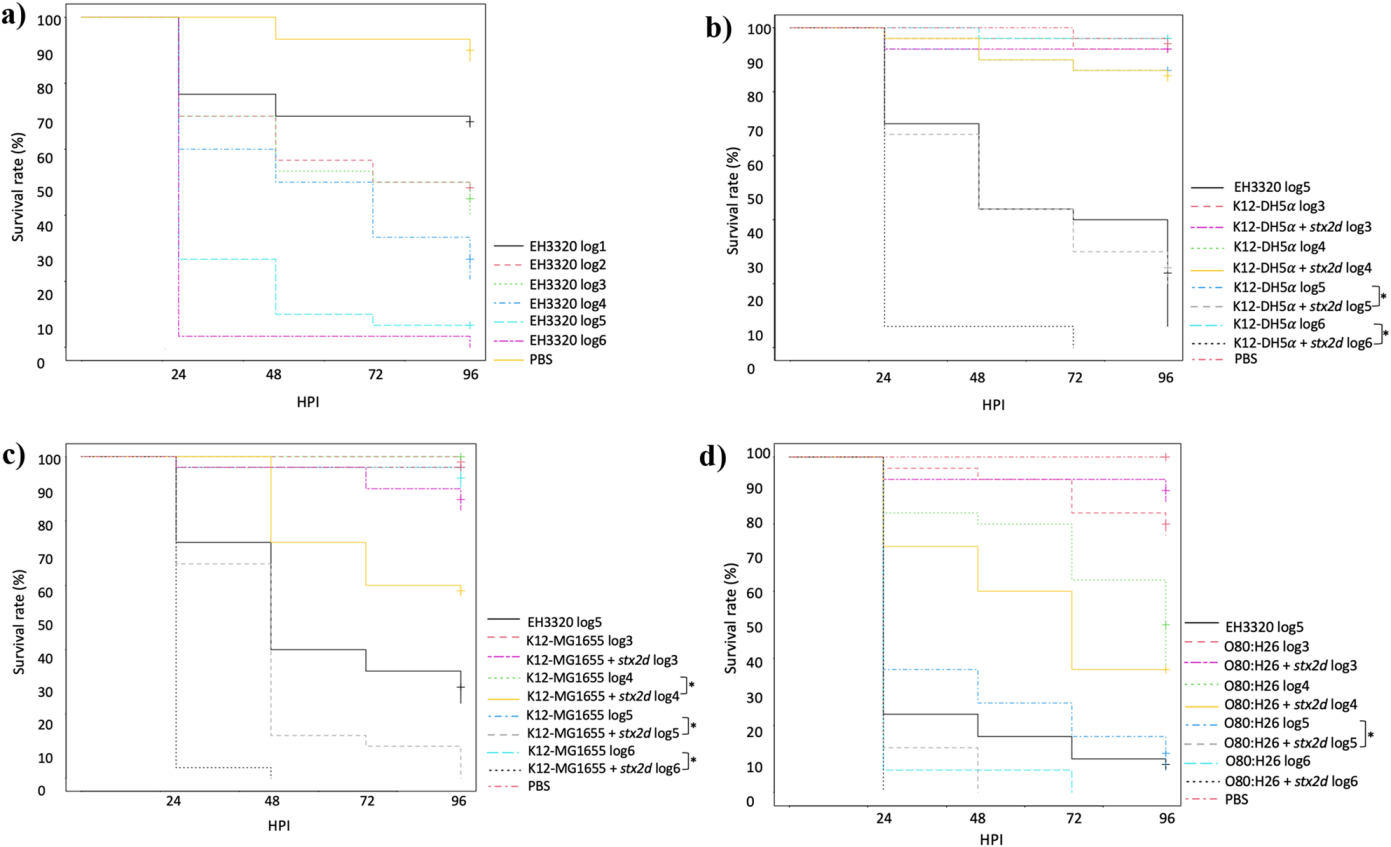
Kaplan–Meier survival curves of the experiments with *G. mellonella* larvae inoculated with (**a**) EH3320 at different concentrations (**b**) K12-DH5α with or without vB_EcoS_ULI-O80_Stx2d (**c**) K12-MG1655 with or without vB_EcoS_ULI-O80_Stx2d (**d**) O80:H26 with or without vB_EcoS_ULI-O80_Stx2d. Each group contained 30 larvae separated in three groups of 10 larvae. *HPI* hours post inoculation. *p < 0.05.

To assess the virulence of the *stx2d* gene, *G. mellonella* larvae were inoculated with transduced and host bacterial strains. The optimal inoculation dose for the strain EH3320 causing a lethality between 90 and 100% after 4 days was 10^5^ CFU/10 µL. The larvae survival rates were lower in the infected groups compared to the PBS control group [0–60% vs. 90% of survival at 96 h post infection (HPI)] (Fig. 5a). The non-converted strain K12-DH5α showed a larvae mortality < 10% for all concentrations, whereas converted K12-DH5α strain showed a larvae mortality of 60–70% in 10^5^ CFU/10 µL after 4 days and of 90% at 10^6^ CFU/10 µL after 1 day (Fig. 5b). For the non-converted K12-MG1655 strain, the larvae mortality was also below 10% for all concentrations, whereas the converted strain showed a larvae mortality rate of 90–100% after 4 days at 10^5^ CFU/10 µL and of 90–100% after 1 day at 10^6^ CFU/10 µL (Fig. 5c). Converted O80:H26 strain showed larvae mortality of 100% after 2 days (Fig. 5d).

## Discussion

Lysogenic phage conversion constitutes an efficient mechanism for rapid dissemination of phage-encoded virulence genes^32^. While in vitro phage-mediated transmission of the *stx2* gene between different laboratory bacterial strains and *E. coli* O157:H7 has already been described^33^, studies providing information on transduction in the O80:H26 strain and the identification of the insertion sites of this *stx2d* gene were lacking. The choice to limit this study to the phage encoding Stx2d was due to the observation that this toxin type is associated with the most severe forms of the infections in humans^34^.

After being challenged with Stx2d phage stock solutions, the K12-MG1655, K12-DHα5, O80:H2 and O80:H26 *E. coli* strains produced lysis areas, but the O18-K1 and ATCC 25922 *E. coli* strains failed to produce any visible lysis. Many factors may contribute to the inability of this phage to infect these bacterial strains, including the lack of the correct receptor for the Stx2d phages or the presence of glucose moieties instead of galactose residues in the terminal position of cell wall lipopolysaccharides^35^. Systems such as Crispr-Cas9^36^ or BREX could also inhibit the phage infection^37^.

The stability of transduced *stx* genes based on the ability of the lysogenized strains to retain the acquired genes up to three subculturing steps in LB broth was determined. The phage Stx2d from the EH3320 strain was stably maintained in all lysogenized hosts as confirmed by PCR. Conversely, the Stx2d phages originating from EH3155 and EH3160 *E. coli* strains were less stable and appeared to be carried transiently in subcultured cells consistent with what was already described by Tozzoli et al.^38^.

Several non-O157 AE-STEC serogroups are important causes of human disease, one of the most common being O80:H2, for which hypotheses of stx genes transduction were already discussed in the literature^39,40^. This study demonstrates that *stx2d*-harboring phages from AE-STEC O80:H2 can transduce EPEC O80:H26 and to non-pathogenic strains, and convert them to stable lysogens unlike the O80:H2 strain. Even if this variant of O80:H2 strain is phylogenetically related to the donor strains^23^, it is possible that strain O80:H2 could contain active defense systems like nucleic acid restriction, abortive infection or even chemical defense^41^. Conversely, O80 STEC can lose *stx* genes at appreciable frequencies, thereby reverting to being EPEC as showed in this study. STEC O80 and EPEC O80 represent a dynamic system in which bidirectional conversion yields different pathotypes. Stx-encoding bacteriophages are the major elements facilitating this conversion. The ability of STEC O80 strains to cause HUS indicates that such organisms are more virulent than EPEC strains lacking the *stx* gene.

If this is true, a change in the pathotype of the infecting *E. coli* O80 strain during an infection by the lost or acquisition of an Stx-encoding bacteriophage might have clinical implications. For instance, using the paradigm that Stx causes HUS, the loss of a *stx* gene during early infection, before Stx production, might prevent the development of HUS for the patient.

Genome analysis revealed that the phage belongs to a currently unclassified genus and family within the *Caudoviricetes* class. It shares 98% identity with Stx2_112808 phage (isolated from an *E. coli* O145:H28 strain isolated in Japan and represent 20.3% of HUS cases in Argentina^42^) and > 95% identity with seven other phages (Supplementary Fig. 1). Homology detection and structure prediction showed that the phage encodes different lysogenic cycle-associated proteins like an integrase, an excisionase, the different repressors CI, CII and the antirepressor Cro. The integrase gene plays an important role in the lysogenic cycle of temperate phages^43^. It is an enzyme associated with phage genome insertion within the bacterial chromosomes at certain attachment sites (attP and attB) by a site-specific recombination process^44^. Shiga toxin containing prophages can convert the host strain since they integrate their genome using specific gene insertion sites. In these three transduced strains, the encoded gene present was the unknown function *yecE*, which is consistent with results from Nyambe et al.^45,46^. A previous study indicated that the genetic diversity of the integrase-encoding genes among various Stx prophages is related to the different insertion sites of these prophages within the bacterial chromosome^47^. While multiple integration sites for Stx phage and formation of double lysogens occurs often^48^, it was not the case in this study. The excisionase controls integrase-mediated DNA rearrangements. It is strongly associated with the Stx prophage regulation induction process, triggering a lytic cycle of Stx prophages to lyse bacterial host and release Stx-converting phages in the environment^3,33^. The different repressors enable the entry of the phage into the lysogenic cycle. The *nanS-p* gene present in the Stx region is the phage homologue of the bacterial gene *nanS*, which encodes an esterase involved in the metabolization of sialic acids produced by submaxillary glands of bovines and present in great quantities in their gastrointestinal tract^49^.

In vivo experiments with *G. mellonella* larvae showed decreased survival rates in larvae treated with the transduced *E. coli* strains. Indeed, only a few hours after bacterial inoculation, some larvae already showed visible signs of infection (decreased activity, melanization). The bacterial dose used (10^6^ CFU/larvae) resulted in a very high mortality within 24 HPI in the infected group. In vivo experiments support the in vitro results with the fact that the phage Stx2 harboring *stx2* genes bring at least a part of the virulence. The difference is less obvious between the two O80 *E. coli* strains because the EPEC O80:H26 *E. coli* strain already possess virulence factors like intimine (*eae*), enterohemolysin (*hly*) and genes present on the pS88 plasmid (*iron, iss, iha*).

In conclusion, the vB_EcoS_ULI-O80_Stx2d phage is stable and can resist to moderate pH and temperature conditions, which may be interesting for its survival in various environments. Our data demonstrate that UV radiation cause induction of lambdoid prophages carrying *stx* genes which can convert non-pathogenic *E. coli* into STECs. This result is in line with the hypothesis that EPEC O80:H2 can become AE-STEC by acquisition of the *stx* phage presented in a recent study^23^.

These findings provide insights into the role of Stx-converting phage in the bacterial pathogenicity evolution through lysogenization in STEC strains. The consequences of this HGT over extended periods of time (frequency of gene acquisition upon transduction, gene stability) may result in a great clinical concern. Moreover, this study showed that newly converted strains are significantly more virulent than the non-converted ones suggesting the important role of the Stx2d phage in the pathogenicity.

Further studies are needed to analyze the Stx production of newly converted STEC and to attempt the transduction between different serotypes strains. Comparison of the genome structure and morphology of all Stx2d phages and *stx2d* genes would allow a better understanding of their epidemiology in the different STEC serotypes. Additional studies are needed to confirm the predicted protein functions of the Stx-convertig phages and the coevolution of Stx-converting phages with their STEC hosts.

## Materials and methods

### Bacterial strains

The three *stx2d* positive EH3155 (SAMN14087149), EH3160 (SAMN14087151) and EH3320 (SAMN14087146) strains used in this study are listed in Table 1. These three strains are phylogenetically very close based on previous work^23^ and also produce the AE lesion. They were provided by ARSIA (Regional Association for Animal Health and Identification) and were used as the donor strains for the phage transduction experiments. Recipient strains for lysogenization experiments were selected based on their avirulent background (*E. coli* K12-MG1655, *E. coli* K12-DH5α, *E. coli* ATCC O18-K1 and *E. coli* ATCC 25922) and their seropathotype (EPEC *E. coli* O80:H26 and EPEC *E. coli* O80:H2).

Strains were routinely grown overnight in Lysogeny Broth (LB) broth at 37 °C with shaking at 150 rpm or on LB agar plates. Prior to infection experiments, the absence and the presence of *stx2d* was verified by colony PCR in the host and donor strains, respectively.

### Induction of Stx2d phages

*Escherichia coli* EH3155, EH3160 and EH3320 (harboring Stx2d encoding prophage) were grown from frozen cryogenic vial stocks, streaked on LB agar plates, grown from single colonies overnight at 37 °C in LB broth and then 1:100 was inoculated into fresh LB medium. The inoculum was incubated at 37 °C, 250 rpm to an OD_600nm_ of 0.6. For UV induction, 10 mL of the three STEC strains cultures were placed in a petri dish with open lid and exposed for 1 min to UV radiation (0.5 kJ/m^2^). Following UV radiation, cultures were maintained for 6 h at 37 °C with gentle shaking. Then, the cultures were centrifuged (10 min at 10,000×*g*) and the supernatants were filtered through a sterile 0.22 μm filter (VWR international) to completely remove bacteria^33^. The three phages were stored at 4 °C.

### Host range and infectivity of Stx2d phages

A total of 200 μL aliquots of the exponential phase (OD_600_ ≅ 0.3) cultures of each host strain were mixed with 100 μL of phage, incubated at 37 °C for 20 min and 2 mL molten soft LB agar was added and poured onto LB Lennox agar plates, then allowed to solidify at room temperature (RT). Plates were incubated for 24 h at 37 °C. If clear lysis zones were observed, results were considered as positive for each Stx2d-phage stock solution.

### Horizontal transfer of *stx2d* gene from O80:H2 *E. coli* to non-pathogenic *E. coli*

*Escherichia coli* strains presenting a clear lysis were grown at 37 °C in 3 mL of LB broth to the early exponential phase, from which 100 µL aliquots were mixed with 3 mL of LB broth tubes containing 100 µL of 1 M CaCl_2_ and 500 µL of each phage stock and incubated overnight at 37 °C without shaking. Infected cells were recovered by centrifugation at 6.000×*g* (20 min, 4 °C) and cell pellets were washed three times with phosphate buffer saline (PBS) to remove non-specifically bound phage particles^50^. The washed pellets were then suspended in 1 mL of LB broth, spread onto STEC agar plates and incubated overnight at 37 °C. A colony from each plate was randomly selected and subcultured three times on STEC agar to identify stable lysogenic bacteria. After DNA extraction, a *stx2d* specific PCR^51^ was performed to confirm whether the converted cells acquired the gene. Stock cultures were prepared of each strain by centrifugation (8000×*g*, 10 min). The pellets were suspended in 1 mL of LB broth and 0.5 mL was transferred to 2 mL beads-containing cryogenic vials and stored at − 80 °C for further use.

### pH and temperature stabilities

To investigate the phage stability at high temperature, 1 mL of 10^8^ PFU/mL of filtrate phage solution was added to a 1.5 mL microtube and incubated at 4 different temperatures (25 °C, 37 °C, 45 °C and 60 °C) for 1 h using a heat block. For pH stability, 100 µL of the phage solution (10^8^ PFU/mL) was mixed with 900 µL PBS adjusted to pH 2–4–6–8–10 and 12 with HCl and NaOH and incubated at room temperature for 1 h. For all conditions, DNA was extracted with the DNA blood and tissue kit (Qiagen) and a qPCR was realized to compare the genomic copy numbers of each sample^52^. After the investigation of the normality of each distribution (corresponding to the concentration data of the phage and for both incubation condition), (by a histogram, a quantile–quantile plot (QQ-plot), a boxplot, and a Shapiro–Wilk test) a one-way analysis of variance (ANOVA) (with multiple comparisons) was used to assess if the concentration obtained after 1 h incubation was significantly different from the original. All statistical analyses were performed using R (*p* value ≤ 0.05)^53^.

### STX phage quantification

A quantitative PCR was performed using a customized *stx2d* sequence as a standard curve: -TCAGGCAGATACAGAGAGAATTTCGTCAGGCACTGTCTGAAACTGCTCCTGTGTATACGTGACGCCGG- (Eurogentec).

The qPCR assays were carried out in a 25 µL volume containing 1 × GoTaq® Master Mix 2.0 (Promega), primers and probe (final concentration 300 nM of each primer and 100 nM probe; Eurogentec) (Table 2) and 5 µL template DNA. qPCR was performed with the following amplification program: initial activation of the enzyme at 95 °C for 5 min followed by 40 cycles of 95 °C for 15 s, then 1 min annealing and elongation at 60 °C and cooling at 40 °C for 30 s^54^.

**Table 2:** Primers and probes for qPCR quantification of *stx2d*^55^.

Gene	Primer or probe	Sequence (5′–3′)	Position (5′–3′)	Accession number
*stx2*	stx2-F	TCA GGC AIA TAC AGA GAG AAT TTC G	578–602	AY443044
	stx2-R_a_	CCG GIG TCA TCG TAT ACA CAG	646–626	AY443044
	stx2-PΦ	CAC TGT CTG AAA CTG CT	608–624	AY443044

^Ф^ Probe tagged with minor groove-binding non-fluorescent quencher (MGBNFQ) and 6-carboxyfluorescin (FAM) fluorescent label (Eurogentec).

### Whole genome sequencing and genomic analysis

The phage genomic DNA was isolated using a phenol–chloroform extraction^55^. Sequencing was performed on an Illumina (San Diego, CA, USA) MiniSeq machine using the Nextera Flex DNA library kit (Illumina). After assembly of the raw sequencing data using Unicycler^56^, the most related phages were identified with BLASTn^57^ and Viptree v3.0^58^. VIRIDIC^59^ was used for taxonomic classification. Annotation was performed with RASTtk^60^ on the PATRIC server^61^ followed by a manual curation using BLASTp and HHPred^62^ and visualization with Easyfig^63^. The data were submitted to NCBI GenBank under accession number ON416862. For the bacterial samples, total genomic DNA were extracted with the DNA blood and tissue kit (Qiagen). The DNA was subsequently prepared for Illumina sequencing similarly to the phage DNA and sequenced. In parallel, the DNA were also prepared for long read sequencing using the Rapid barcoding kit (Oxford Nanopore Technology, Oxford, UK) and sequenced on a MinION R9.4.1 flowcell (Oxford Nanopore Technology), with Guppy(v3.1.5) as basecaller^64^. The genomes were also constructed using Unicycler. The assemblies were visually inspected using Bandage^65^. A quality control with Quast^66^ revealed an N50 of 5,264,684 bp (13 contigs), 3,013,105 bp (43 contigs) and 3,012,995 bp (55 contigs) for K12-MG1655, K12-DH5α and O80:H26, respectively.

### Transmission electron microscopy

*Escherichia coli* EH3320 was grown overnight at 37 °C in LB broth. The culture was subsequently diluted 50× in 50 mL of LB broth. Phage induction was carried out by adding mitomycin C (concentration of 1.0 μg/mL) to the culture bottles at OD_600_ ≅ 0.2, which were further incubated until an OD decrease_._ The culture was centrifuged at 10,000×*g* for 10 min at 4 °C. The phage was negatively stained and analyzed by transmission electron microscopy (TEM) by the Electron Microscopy unit (ULB, CMMI, Gosselies, Belgium). The grids were stained with 4% uranyl acetate. The sample was placed on the grid using the grid-on-drop method. Observations were made on a Tecnai10 TEM (FEI-TechnoFisher) and images were captured with a Veleta CCD cam.

### In vivo assay: *Galleria mellonella* larvae model

A preliminary experiment aimed to determine the optimal inoculation dose was realized. Six groups of 10 *G. mellonella* larvae (Animal Confort, Loncin, Belgium) were inoculated using an automatic injector (Cole Parmer, Vernon Hills, IL, USA) with 10 µL of EH3320 at six different concentrations, ranging from 10 to 10^6^ CFU/10 µL. Each larva was inoculated in the last left proleg with a BD Plastipak™ 1 mL sterile syringe (Becton–Dickinson, Franklin Lakes, NJ, USA) and a sterile 30-gauge needle (Terumo corporation, Tokyo, Japan). The optimal inoculation dose was expected to cause a lethality of 90–100% after 4 days.

The principal experiments were carried out in technical triplicate to compare the survival of *G. mellonella* larvae with the non-STEC strains converted or not with the Stx2d phage. For each experiment (three transduced strains), 270 larvae were divided into nine groups (Table 3). Each larvae was inoculated as previously described. The larvae were incubated at 37 °C and mortality was evaluated every 24 h. Kaplan–Meier survival curves were generated to assess the survival of the different groups using R-commander (Rcmdr v2.6-0). Logrank tests were performed to highlight any significant difference in survival rates between the groups (*p* < 0.05). Back-titration of bacteria was realized to verify the inoculated doses.

**Table 3:** Summary table of the *G. mellonella* larvae groups inoculated in triplicate for the main experiments (survival and titration).

Groups	Experiments		
	1 (CFU/10 µL)	2 (CFU/10 µL)	3 (CFU/10 µL)
1	PBS	PBS	PBS
2	K12-DH5α (10^3^)	K12-MG1655 (10^3^)	O80:H26 (10^3^)
3	K12-DH5α (10^4^)	K12-MG1655 (10^4^)	O80:H26 (10^4^)
4	K12-DH5α (10^5^)	K12-MG1655 (10^5^)	O80:H26 (10^5^)
5	K12-DH5α (10^6^)	K12-MG1655 (10^6^)	O80:H26 (10^6^)
6	K12-DH5α + *stx2d* (10^3^)	K12-MG1655 + *stx2d* (10^3^)	O80:H26 + *stx2d* (10^3^)
7	K12-DH5α + *stx2d* (10^4^)	K12-MG1655 + *stx2d* (10^4^)	O80:H26 + *stx2d* (10^4^)
8	K12-DH5α + *stx2d* (10^5^)	K12-MG1655 + *stx2d* (10^5^)	O80:H26 + *stx2d* (10^5^)
9	K12-DH5α + *stx2d* (10^6^)	K12-MG1655 + *stx2d* (10^6^)	O80:H26 + *stx2d* (10^6^)

## Supplementary Information

Supplementary Figure S1.

## Data Availability

The data were submitted to NCBI GenBank under accession number ON416862.
