# Supplementary figures and images for: Impact of Shiga-toxin encoding gene transduction from O80:H2 Shiga toxigenic Escherichia coli (STEC) on non-STEC strains
Source: Sci Rep. 2022 Dec 14;12:21587. doi: 10.1038/s41598-022-26198-8 (PMC9751135; doi:10.1038/s41598-022-26198-8)

Figure S1: Intergenomic distance between vB\_EcoS\_ULI-O80\_Stx2d phage and related bacteriophages.

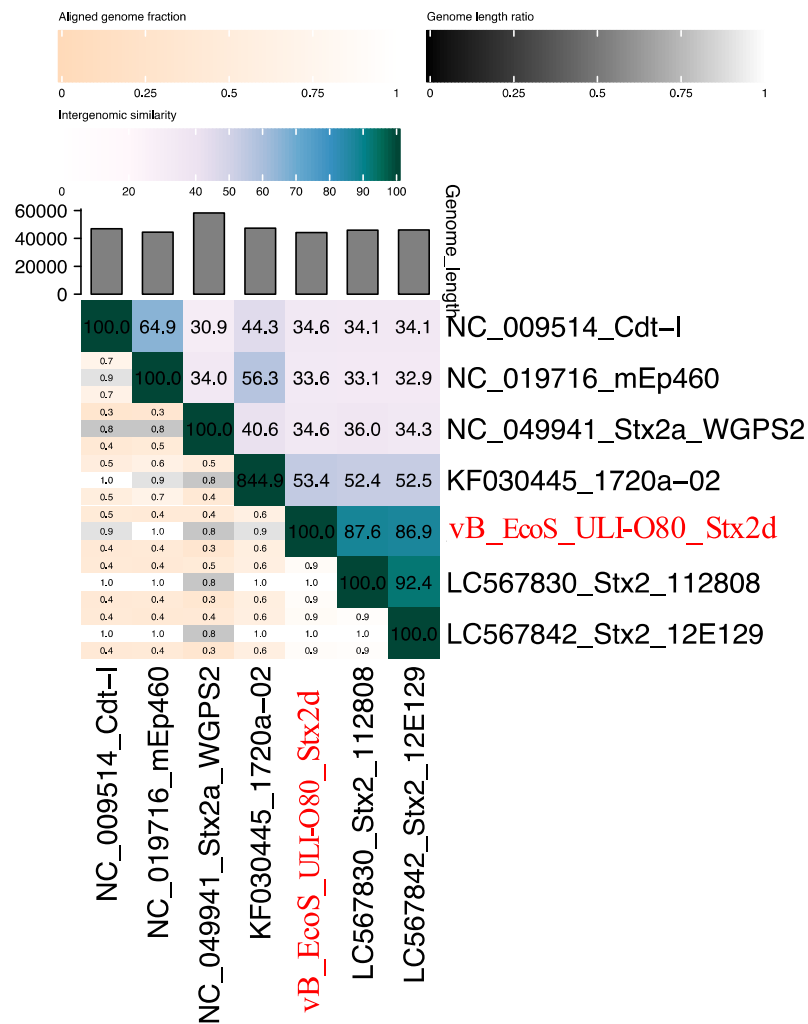

Supplement: Supplementary file 1 — Supplementary Figure S1. [file 41598_2022_26198_MOESM1_ESM.pdf]
